# The effect of ultrapro or prolene mesh on postoperative pain and well-being following endoscopic Totally Extraperitoneal (TEP) hernia repair (TULP): study protocol for a randomized controlled trial

**DOI:** 10.1186/1745-6215-13-76

**Published:** 2012-06-07

**Authors:** Nelleke Schouten, Thijs van Dalen, Niels Smakman, Sjoerd G Elias, Geert Jan Clevers, Egbert-Jan M M Verleisdonk, Paul H P Davids, Ine PJ Burgmans

**Affiliations:** 1 Department of Surgery/Hernia Clinic, Diakonessenhuis, Utrecht, The Netherlands; 2 Julius Center for Health Sciences and Primary Care, Utrecht, The Netherlands; 3 Department of Surgery/Hernia Clinic, Diakonessenhuis, Room: Secretariaat Heelkunde, Professor Lorentzlaan 76, 3707 HL, Zeist, The Netherlands

**Keywords:** Endoscopic hernia repair, TEP, Mesh, Chronic postoperative pain, Quality of life

## Abstract

**Background:**

The purpose of this study was to describe the rationale and design of a randomized controlled trial analyzing the effects of mesh type (Ultrapro *versus* Prolene mesh) on postoperative pain and well-being following an endoscopic Totally Extraperitoneal (TEP) repair for inguinal hernias (short: TULP trial).

**Methods and design:**

The TULP trial is a prospective, two arm, double blind, randomized controlled trial to assess chronic postoperative pain and quality of life following implantation of a lightweight (Ultrapro) and heavyweight (Prolene) mesh in endoscopic TEP hernia repair. The setting is a high-volume single center hospital, specializing in TEP hernia repair. All patients are operated on by one of four surgeons. Adult male patients (≥18 years of age) with primary, reducible, unilateral inguinal hernias and no contraindications for TEP repair are eligible for inclusion in the study. The primary outcome is substantial chronic postoperative pain, defined as moderate to severe pain persisting ≥ 3 months postoperatively (Numerical Rating Scale, NRS 4–10). Secondary endpoints are the individual development of pain until three years after the TEP procedure, the quality of life (QoL), recurrence rate, patient satisfaction and complications.

**Discussion:**

Large prospective randomized controlled studies with a long follow-up evaluating the incidence of chronic postoperative pain following implantation of lightweight and heavyweight mesh in endoscopic (TEP) hernia repair are limited. By studying the presence of pain and quality of life, but also complications and recurrences in a large patient population, a complete efficiency and feasibility assessment of both mesh types in TEP hernia repair will be performed.

**Trial registration:**

The TULP study is registered in the Dutch Trial Register (NTR2131)

## Background

Inguinal hernia repair is one of the most common surgical procedures worldwide. In The Netherlands, approximately 30,000 hernia repairs are performed annually. The lifetime risk of undergoing a hernia operation is 27 % for men and 3 % for women [1]. The incidence of hernia recurrence has been the primary endpoint in inguinal hernia studies for many years, but with the introduction of the tension-free mesh repair, recurrence rates have dropped significantly to 2 to 3 % [2]. More recently, attention is being paid to several quality of life (QoL) aspects, with time to full recovery and chronic postoperative pain being addressed in particular [1,3].

Chronic pain after inguinal hernia surgery is a common complication. The incidence of chronic postoperative pain after inguinal hernia repair, as described in the literature, varies significantly (10 to 30 %), which is partially explained by the lack of a clear definition [1-4]. The effect of pain on quality of life is significant, since functional limitations of pain in daily life activities are experienced by 2 % to 20 % of the patients [5].

In experienced hands, endoscopic hernia repair techniques are associated with significantly less postoperative pain and an earlier return to normal activities compared to conventional (Lichtenstein) hernia repair [4,6,7]. Totally Extraperitoneal (TEP) is preferred over Transabdominal Preperitoneal (TAPP) hernia repair, since it is less invasive and associated with fewer visceral injuries [2,8]. In addition, recent studies, (in patients treated with a Lichtenstein or TEP procedure), suggest that lightweight meshes may be associated with less chronic postoperative pain compared to heavyweight meshes [9-12]; the latter create maximum mechanical stability and promote the formation of scar tissue, while the properties of the lightweight mesh aim to improve integration in the abdominal wall and prevent the formation of scar tissue [13,14]. There seems to be no (significant) difference in the risk of a recurrent groin hernia between a lightweight and heavyweight mesh [9,12,15].

In addition, since TEP is associated with less chronic postoperative pain compared to a conventional repair (Lichtenstein), an endoscopic TEP hernia repair with implantation of a lightweight mesh could, therefore, be an appealing technique in the prevention of chronic postoperative pain after groin hernia surgery.

The aim of this manuscript is to describe the rationale and design of a large, prospective, double blind, randomized clinical study comparing the results of chronic postoperative pain and quality of life following endoscopic TEP hernia repair with a heavyweight (Prolene) and a lightweight mesh (Ultrapro).

## Methods and design

### Study design

**Figure 1:**
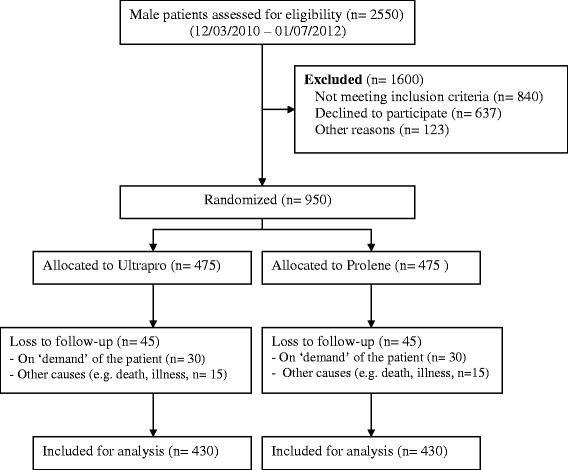
CONSORT flow-diagram, estimated recruitment targets.

This is a prospective, double blind, randomized clinical trial involving a high-volume hospital in The Netherlands specialized in the TEP technique for inguinal hernia repair (Diakonessenhuis, Utrecht/Zeist, The Netherlands). All hernia operations are performed by four experienced TEP surgeons. Adult (≥18 years) male patients with primary, unilateral inguinal hernias, eligible for TEP hernia repair, are allocated to either lightweight mesh (Ultrapro, Ethicon, a Johnson & Johnson company, Amersfoort, The Netherlands) or heavyweight mesh (Prolene, Ethicon, a Johnson & Johnson company, Amersfoort, The Netherlands) by randomization. The total follow-up of patients is three years. A flowchart of the study with the estimated recruitment targets is shown in Figure 1. The design, conduct and reporting of this study will adhere to the Consolidated Standards of Reporting Trials (CONSORT) guidelines [16].

### Patient population

A total number of 950 patients will be included in the trial (see “Sample size considerations”). Patients are recruited during their first visit at the outpatient clinic of the participating hospital. Patients are screened for eligibility according to the criteria listed in Table 1. Male patients ≥18 years of age with primary, reducible, unilateral inguinal hernias and no contraindications for endoscopic TEP repair are eligible for inclusion in the study. Based on a previously conducted cohort study, the estimated mean age of patients is 53 (SD ± 14) years, the proportion of patients with a job is approximately 85 % and over 90 % of the patients have an American Society of Anesthesiologists (ASA) 1 or 2 score.

**Table 1:** Inclusion and exclusion criteria

Inclusion criteria:	Exclusion criteria:
- Male gender	- Female gender
- Age ≥18 years	- Bilateral hernia
- Primary, unilateral, reducible inguinal hernia	- Scrotal hernia - Recurrent hernia
- Eligible for TEP (and general anesthesia)	- Strangulated hernias (necessitating emergency hernia repair)
	- Collagen or connective tissue disorders, such as Marfan’s syndrome
	- Likely problems with maintaining follow-up (for example, patients with no fixed address or insufficient comprehension of Dutch language

### Intake

The study information is sent to patients after an appointment is made for an initial consultation. The appointment with one of four surgeons specialized in TEP hernia repair is scheduled within one week. After screening for eligibility criteria, informed consent is obtained and the patient is included in the study. Preoperative patient data are obtained by the coordinating investigator (Table 2).

**Table 2:** Preoperative data

- Age	- Pain:^*^
- ASA-classification	· NRS score
- Co-morbidity (for example other pain syndromes)	· Brief Pain Inventory (BPI)
- Medication	· Inguinal Pain Questionnaire (IPQ)
- Smoking	· Carolinas Comfort Scale (CCS)
- Body Mass Index (BMI) - Education and occupation	· Pain-related Sexual Function Questionnaire (PSF)
- Health-related Quality of Life:^*^	
· SF-36 Questionnaire	
· EQ-5D Questionnaire	

* see section “Follow-up” of the manuscript (pages 7–8).

### Randomization

Patients are randomly assigned to either Ultrapro or Prolene mesh in the operating room (after patients are under general anesthesia) by computerized block randomization. Follow-up of patients will take place according to the intention-to-treat principle. The patient, coordinating investigator and the surgeon involved in the follow-up of enrolled patients are blinded for the allocated mesh. Obviously, the operating surgeon cannot be blinded to the study because of the characteristic composition of the two meshes. To avoid bias, the mesh type is not mentioned in the operating chart. In addition, the follow-up is not by the operating surgeon, but by one of the other three surgeons specialized in hernia repair.

### Intervention

The perioperative care and surgical technique do not differ between the study groups and are not different from the standard procedure. There are no other interventions than the implantation of a lightweight mesh (Ultrapro) or a heavyweight mesh (Prolene) during the TEP endoscopic procedure. General anesthesia is applied in all patients. The operative details of the TEP technique have been described previously [7,17]. The mesh graft is not fixated; it reduces operative time, saves costs and avoids possible entrapment neuralgia. Hernia types are classified during TEP repair according to the Nyhus classification. Intra-operative complications and operative time are registered in the Electronic Patient Chart (Dutch: EPD).

#### *Prolene*

Prolene® is a non-soluble, so-called heavyweight mesh.[14] It is the mesh commonly used in endoscopic TEP hernia repair in our hospital. The mesh characteristics are:

Structure: Monofilament with small pores

Polymer: Polypropylene

Weight: 80 to 85 g/m^2^

Dimensions: 10 x 15 cm

#### *Ultrapro*

Ultrapro® is a lightweight mesh [14]; the monocryl (polyglecapron) is absorbed within 90 to 120 days due to hydrolysis, leaving a lightweight mesh with a pore size of 3 to 4 mm. The mesh characteristics are: 

Structure: Monofilament with large pores (3 to 4 mm)

Polymer: Polypropylene (PP) and Monocryl (Poliglecapron)

Weight: 28 g/m^2^ (part of the polypropylene that is not absorbed

Dimensions: 10 x 15 cm

### Postoperative management

Patients are discharged on the day of surgery, unless complications occur. The duration of hospital stay and postoperative complications are registered in the EPD. At discharge, patients are advised to take analgesics (Paracetamol and, if necessary, Diclofenac), when necessary and avoid strenuous physical activity (lifting, sports) during the first post-operative week. There are no other (physical) restrictions. The type and amount of analgesics used during the first postoperative week are kept in a pain diary.

### Follow-up

Several questionnaires are used for the assessment of pain and quality of life (also to evaluate which questionnaire can best be used to evaluate pain and its effect on quality of life in this patient population).

1) To assess the presence of postoperative pain, the following validated questionnaires are used:

Inguinal Pain Questionnaire (IPQ, Dutch version) [18]

Brief Pain Inventory, Short Form (BP-SF, Dutch version) [19,20]

Carolinas Comfort Scale (CCS, Dutch version) [21]

Numerical Rating Scale (NRS, 0 = no pain, 10 = extremely painful, Dutch version)

2) To measure the health related Quality of Life (QoL) the following validated questionnaires are used:

Short Form (36) Health Survey (SF-36, Dutch version) [22]

EuroQol 5D (EQ-5D, Dutch version) [23]

3) Finally, to assess the presence of pain related to sexual function, the PSF (pain related to sexual function) questionnaire is used, which is a Dutch translation of the questionnaire similar to the one previously described by Aasvang *et al.*[24] and based on the International Index of Erectile Function (IIEF) [25] to assess the presence, frequency, intensity and location of pain during sexual activity and the effect of pain on sexual function.

The patient is requested to record – on a daily basis during the first week immediately following surgery – the pain experienced, as well as the type and amount of analgesics used.

The NRS is used to assess the amount of experienced pain, where 0 = no pain and 10 = extremely painful. BPI and SF-36 questionnaires are filled in at Day 1 and Day 7 following surgery, to determine the effect of pain on daily activities.

After 6 weeks and 3, 12, 24 and 36 months, the IPQ, BPI, CCS, NRS, PSF, SF-36 and EQ-5D questionnaires are completed by the patients. To prevent bias, the questionnaires will be completed at home, or at the visit to the outpatient clinic at three months and one year postoperatively, in the absence of the surgeon or coordinating investigator, before the clinical assessment at the outpatient clinic. Patient satisfaction with the operative procedure is assessed at one week and three months after surgery on a 0 (= very unsatisfactory) to 10 (= very satisfactory) Likert scale.

All patients are examined in the outpatient department by one of the four surgeons (different from the operating surgeon) and the coordinating investigator at three months and one year after surgery. The physical examination is, if necessary, supplemented with an Ultrasound or MRI scan in patients with groin pain and/or complaints suggestive of a recurrent hernia.

All visits include standardized clinical evaluation (according to the validated ‘Inguinal Pain Form (Dutch ‘Liespijnformulier’) by Loos [26]) and registration of possible complications. Parameters assessed at the outpatient evaluation are:

Complications after surgery

Short term (for example: seroma, hematoma, wound-infection, urinary retention, constipation)

Long term (for example: testicular atrophy, mesh infection)

The presence of a recurrent hernia

The presence of a contra-lateral hernia

Other non-hernia related problems in the groin region (for example, hydrocele, varicocele, prostate problems/hidradenitis)

Postoperative pain

Characteristics of pain

The relation of pain to daily activities, physical activity and/or sexual activity

Possible treatment of pain

Mesh feeling

Sensibility disorders in the inguinal/scrotal/thigh/umbilical region or elsewhere

### End-points

The primary endpoint is the presence of moderate to severe chronic pain three months after an endoscopic preperitoneal (TEP) hernia correction, as measured by the NRS. The definition of the International Association for the Study of PAIN (IASP) is used with chronic postoperative pain being defined as pain still present at the site of the operation three months after the primary surgery and different from the pain before the operation. Pain is measured on a 0 (= no pain) to 10 (extremely painful) scale. In accordance with the literature, the following cut-off values for pain are used: [26]

Mild pain: NRS 1 to 3

Moderate pain: NRS 4 to 6

Severe pain: NRS 7 to 10

Moderate to severe pain is considered as clinically relevant [26]. Only moderate to severe pain (NRS 4 to 10) will, therefore, represent ‘chronic pain’ in this study. Secondary endpoints are:

Duration of hospital stay

Complications: intra-operative, post-operative period (until three months after surgery), long-term (until three years after surgery, for example, testicular atrophy)

Time to resume daily activities and return to work

Recurrent hernia

Contra-lateral hernia

Development of pain over time as measured by the NRS score at the first week following TEP repair and 6 weeks, 12, 24 and 36 months postoperatively

Development of pain over time as measured by the BPI, IPQ and CCS questionnaires: the presence of pain in the first week postoperatively and after 6 weeks, 3 months, 12, 24 and 36 months

Development of pain, sensitivity disorders (anesthesia, hypoesthesia, hyperesthesia, allodynia) and or discomfort (including sensation of ‘mesh presence’ at the outpatient visit at three months and one year post-operatively

Health related quality of life as measured by the SF-36 and EQ-5D questionnaires

Pain related sexual function as measured by the PSF questionnaire

Patient satisfaction with the procedure and perioperative care

### Safety measures

Surgeons operating on patients for this study have extensive experience with TEP repair (over 500 procedures per surgeon). Before the start of this study, every surgeon had performed over 20 procedures with an Ultrapro mesh, to prevent the occurrence of a ‘learning curve’ effect with a new mesh in the study period. Serious adverse events (SAE), other than SAEs that might eventually occur following endoscopic TEP repair (hemolytic shock, bladder or bowel injury) are not expected to occur in this trial.

### Sample size and power

The most common definition of chronic postoperative pain is from the International Association for the study of pain (IASP), where chronic pain is defined as pain lasting longer than three months following the primary operation, and other than the pain that was experienced preoperatively. The hypothesis is that the incidence of chronic pain is lower after implantation of a lightweight mesh (Ultrapro) than after implantation of a heavyweight mesh (Prolene). Based on a pilot study in our hosopital the assumption is made that 23% of the patients with a Prolene mesh will experience pain three months after TEP repair. In the Ultrapro group a reduction of 7.5% in the incidence of pain is expected. With a two-sided alpha of 0.05 and a power of 0.80, a total of 429 patients are to be included in each allocation group. Based on previous experience, a loss to follow-up of 10% is expected. Therefore, a total of 950 patients will be included in the TULP trial.

### Statistical methods

Data will be analyzed according to the intention-to-treat principle. SPSS software (SPSS, Chicago, IL, USA) will be used for statistical analysis. Descriptive statistics are used for baseline data. The incidence of the primary endpoint (moderate to severe chronic pain three months after endoscopic TEP repair as based on a NRS 4 to 10) will be compared between the Ultrapro and Prolene mesh group by means of Chi-square analysis. The comparisons of pain will be adjusted for analgesics used as a secondary supporting analysis. The number-needed-to-treat to prevent one case of chronic pain will be calculated. In an additional multiple logistic regression model, the effect of treatment on the primary endpoint will be assessed in relation to important risk factors for pain (younger age, severe pain in the postoperative period and severe preoperative pain [27]). Secondary endpoints will be analyzed by using a Student’s *t*-test (normally distributed continuous), Mann–Whitney analysis (not normally distributed continuous) or Chi-square analysis (categorical variables). The individual development of pain over time will be analyzed with repeated measurement strategies. Effect estimators are described with 95 % confidence intervals. Significance is set at a level of *p ≤ 0.05*.

## Discussion

The TULP trial is a prospective, randomized controlled (RCT) clinical trial of a lightweight mesh (Ultrapro) and a heavyweight mesh (Prolene) in endoscopic TEP hernia repair designed to analyze the outcomes regarding chronic postoperative pain and quality of life.

Although a limited number of randomized studies assessing the outcomes after implantation of a lightweight and heavyweight mesh in endoscopic TEP hernia repair has been published previously, the TULP trial is, to our knowledge, the first randomized controlled trial with a large patient population and a long-term follow-up assessing pain as a primary endpoint and the effect of pain on quality of life. The main challenges that we anticipate are the recruitment and follow-up of trial participants, (following the findings of a previous pilot study patients who visit our hernia clinic are ‘busy’ male patients in the ‘prime of their lives’, who do not always ‘feel like it’ to fill in and return questionnaires). We aim to maximize participant follow-up by face-to-face contact (at three months and one year after TEP repair) and telephone contact as a reminder to return the questionnaires.

There are some limitations of this study. Due to the different characteristics of the meshes, blinding of the operating surgeon is not possible. Nevertheless, the investigator-related bias for the (primary) endpoints is expected to be negligible, since both the coordinating investigator as well as the surgeon involved in the follow-up of the patients, are blinded for the type of mesh. In this study, the same investigator will be present during all patient visits at the outpatient clinic in order to improve consistency of the results and reduce the loss-to-follow-up rates.

In addition, the setting of this study is a high volume center, specialized in endoscopic TEP hernia repair. Despite an inclusive set of patients, a criticism might be that the findings may not be easily generalized to other, less experienced, TEP surgeon settings. On the other hand, the TEP technique has a long(er) learning curve. Expertise is, therefore, a prerequisite in performing TEP and most centers deliver TEP in the hands of expert surgeons, which enables extrapolation of the results of this study.

This prospective, double blind, randomized clinical trial is designed to compare the incidence of chronic postoperative pain following the implantation of heavyweight (Prolene) and lightweight (Ultrapro) mesh in endoscopic TEP hernia repair. As the presence of pain in the groin region, quality of life, return to daily activities and work, patient satisfaction, complications and recurrences are assessed in a large cohort of patients with a sufficient long follow-up period, this study will provide a complete efficiency and feasibility assessment of both meshes in endoscopic TEP hernia repair.

### Trial status

This study has been approved by the regional Medical Ethics Committee (VCMO, Nieuwegein, the Netherlands) and the local Ethics Board of the Diakonessenhuis Utrecht, the Netherlands. This study is performed in accordance with the ethical standards of the Declaration of Helsinki. Recruitment of patients started on 12 March 2010. To date (1 July 2011), 569 patients have been included in the study. Depending on the number of patients needed to be included in the trial (see sample size considerations), recruitment of the 950 patients is currently expected to end in June 2012. Analysis and reporting of data are subsequently expected eight months (outcomes three months after TEP repair) and three and one half years (outcomes three years after TEP repair) later to be complete (October 2012 and October 2015 respectively). The TULP study is registered in the Dutch Trial Register (NTR2131).

## Abbreviations

ASA score: American Society of Anesthesiologists score; BMI: body mass index; BPI: Brief Pain Inventory; BP-SF: Brief Pain Inventory, Short Form; CCS: Carolinas Comfort Scale; CONSORT: Consolidated Standards of Reporting Trials; EPD: electronic patient chart; EQ-5D: EuroQoL 5D; IASP: International Association for the Study of PAIN; IIEF: International Index of Erectile Function; IPQ: Inguinal Pain Questionnaire; NRS: numerical rating scale; PSF: Pain-related Sexual Function Questionnaire; QoL: quality of live; RCT: randomized controlled trial; SAE: serious adverse events; SF-36: Short-form 36; TAPP: Transabdominal preperitoneal; TEP: totally extraperitoneal.

## Competing interests

All authors of this manuscript, and the individuals mentioned in the ‘Acknowledgment’ section, hereby confirm that a Research Grant has been assigned to the Diakonnessenhuis Utrecht/Zeist, or more specifically to the Hernia Centre Zeist, by Johnson & Johnson. The Research Grant is intended to support all (completed) manuscripts on the results and complications of the Totally Extraperitoneal (TEP) endoscopic hernia repair. The Grant will not exceed an amount of € 40.000.

This study itself is not directly the subject of the above mentioned Research Grant or any other financial sponsorship. Johnson & Johnson or any other party has and will have no access to data upon which the manuscript is based. A copy of the manuscript will only be provided at acceptance of the manuscript. Johnson & Johnson has no influence on the (subject of) this study whatsoever. Objectivity of data is, therefore, guaranteed and there is no conflict of interest.

There are no (other) commercial associations that might pose a conflict of interest in connection with the submitted article.

## Authors’ contributions

NS provided the conception and design of the article and contributes in the coordination of the study (as the coordinating investigator). NS and JPJB are responsible for the design of the initial protocol and follow-up of the study. JPJB is also responsible for revising the manuscript critically for important intellectual content. SGE provided the statistical analysis and advice. EJMMV, PHPD and GJC were all responsible for the acquisition of data and critically revising the manuscript. All authors gave final approval of the version to be published.
